# Adolescent suicidal behaviour in Namibia: a cross-sectional study of prevalence and correlates among 3,152 school learners aged 12–17 years

**DOI:** 10.1186/s12888-023-04646-7

**Published:** 2023-03-15

**Authors:** Emmanuel Nii-Boye Quarshie, Nutifafa Eugene Yaw Dey, Kwaku Oppong Asante

**Affiliations:** 1 Department of Psychology, University of Ghana, P.O. Box LG 84, Accra, Ghana; 2 Department of Psychology, University of the Free State, Bloemfontein, South Africa

**Keywords:** Adolescents, Attempted suicide, Global school-based student health survey, Ideation, Namibia, Suicidal behaviour, Suicide

## Abstract

**Background:**

While adolescent suicidal behaviour (ideation, planning, and attempt) remains a global public health concern, available county-specific evidence on the phenomenon from African countries is relatively less than enough. The present study was conducted to estimate the 12-month prevalence and describe some of the associated factors of suicide behaviour among school-going adolescents aged 12–17 years old in Namibia.

**Methods:**

Participants (n = 4531) answered a self-administered anonymous questionnaire developed and validated for the nationally representative Namibia World Health Organization Global School-based Student Health Survey conducted in 2013. We applied univariate, bivariable, and multivariable statistical approaches to the data.

**Results:**

Of the 3,152 analytical sample, 20.2% (95% confidence interval [CI]: 18.3–22.2%) reported suicidal ideation, 25.2% (95% CI: 22.3–28.4%) engaged in suicide planning, and 24.5% (95% CI: 20.9–28.6%) attempted suicide during the previous 12 months. Of those who attempted suicide, 14.6% (95% CI: 12.5–16.9%) reported one-time suicide attempt, and 9.9% (95% CI: 8.1–12.1%) attempted suicide at least twice in the previous 12 months. The final adjusted multivariable models showed physical attack victimisation, bullying victimisation, loneliness, and parental intrusion of privacy as key factors associated with increased likelihood of suicidal ideation, planning, one-time suicide attempt, and repeated attempted suicide. Cannabis use showed the strongest association with increased relative risk of repeated attempted suicide.

**Conclusion:**

The evidence highlights the importance of paying more attention to addressing the mental health needs (including those related to psychological and social wellness) of school-going adolescents in Namibia. While the current study suggests that further research is warranted to explicate the pathways to adolescent suicide in Namibia, identifying and understanding the correlates (at the individual-level, family-level, interpersonal-level, school context and the broader community context) of adolescent suicidal ideations and non-fatal suicidal behaviours are useful for intervention and prevention programmes.

**Supplementary Information:**

The online version contains supplementary material available at 10.1186/s12888-023-04646-7.

## Background

The World Health Organization (WHO) defines suicidal behaviour as “a range of behaviours that include thinking about suicide (or ideation), planning for suicide, attempting suicide and suicide itself” [1]. Suicidal ideation, suicidal planning, and (repeated) attempted suicide represent important risk factors for suicide mortality in the general population [1–3]. Globally, 703,000 suicidal deaths are recorded annually, representing more than one in every 100 deaths in 2019 [4]. Among young persons aged 15–19 years, suicide was the third leading cause of death among girls (after maternal conditions) and the fourth leading cause of death in boys, after tuberculosis – as at the end of 2019 [4]. About 79% of the world’s suicides are recorded in low- and middle-income countries (LMICs), with the Africa region recording the highest rate – 11.2 per 100,000 people [4, 5]. However, national-level representative data on suicidal ideation, planning, and attempts, and their associated factors are still less than enough to support research-informed intervention and prevention efforts and programmes, particularly in LMICs, including those in Africa [1].

Evidence from a recent global systematic review and meta-analysis suggests varying 12-month prevalence estimates of suicidal behaviour among adolescents: suicide ideation = 14.2% (95% confidence interval [CI] = 11.6–17.3%), suicidal planning = 7.5% (CI = 4.5–12.1%), and suicide attempt = 4.5% (95% CI = 3.4–5.9%) [6]. Comparatively, pooled regional rates drawing on published data from the World Health Organization Global School-based Student Health Survey (WHO-GSHS) indicate that the 12-month prevalence estimates of adolescent suicidal behaviour in LMICs are higher in the African region: ideation = 21% (95%CI = 20.1–21.0%), suicidal plan = 23.7% (95%CI = 19.1–28.3%), and attempt = 16.3% (95% CI = 8.4–29%) [7–9]. Notably, the 12-month median prevalence estimate of adolescent self-harm in Southern sub-Saharan Africa – where Namibia is located – (16.5% interquartile range [IQR] = 10.9–24.0%) is comparable to the overall estimate across the sub-Saharan region (16.9% [IQR] = 11.5–25.5%) [10].

Factors associated with suicidal behaviour among adolescents in LMICs, including those in (sub-Saharan) Africa, have been found to exist at the *individual-level* (e.g., [female] gender, low self-worth, hopelessness, HIV/AIDS and other chronic medical conditions in adolescents, alcohol and drug [mis]use, depression, and anxiety and other untreated psychiatric conditions) [7, 8, 10, 11], *family-level* (e.g., parental understanding and support, food insecurity [hunger], conflict with parents, parental divorce, physical abuse victimisation, conflict between parents, child marriage, parental/family poverty) [8, 10, 12], *interpersonal-level* (e.g., lack of peer support, romantic relationships problems, breakups, sexual abuse victimisation) [8, 10, 12], *school context* (bullying victimisation, peer support at school, poor school climate, peer suicide or attempted suicide, and poor academic performance, truancy) [8, 12], and *the broader community-level* – e.g., community violence or war, poverty [10, 12–14]. The multi-layered and multi-contextual nature of the factors associated with adolescent suicidal behaviour can be understood within the socio-ecological model. The socio-ecological model provides a helpful framework to understanding and preventing (adolescent) suicidal behaviour in that the model considers an integration of population-specific and general risks and protective factors [1, 15, 16].

Within Southern sub-Saharan Africa, South Africa remains the only country with relatively large data on adolescent self-harm and suicidal behaviour [10, 17–19]. Hence, towards contributing evidence to addressing the dearth of research on adolescent suicidal behaviour in Southern sub-Saharan African countries (apart from South Africa), several scholars have explored and published evidence from the WHO-GSHS data accessed from some countries in the sub-region: Botswana [20], Eswatini [14], Malawi [21], Mauritius [22], Mozambique [23, 24], Zambia [25], and Zimbabwe [26]. Thus far, it is only the published study by Peltzer and Pengpid (2017) drawing on the Namibian WHO-GSHS data that reports evidence on suicide attempt [27]. In other words, no peer-reviewed publication is available on the secondary analysis of the country-specific prevalence and correlates of suicidal ideation, planning, and (repeated) suicide attempt in the nationally representative school-going adolescents sample (aged 17 years and younger) of the Namibian WHO-GSHS data. It must be noted that the 2013 Namibian WHO-GSHS data is the latest available dataset from the country’s participation in the survey.

Namibia has a youthful population, as persons aged 17 years and younger constitute 43% of the general population [28]. The mean years of schooling in Namibia is 7.2 years [29]. Primary to middle school education, which stretches from grade 1 through 7, is compulsory for all children between the ages of 6 and 16 years, while secondary education remains optional [30]. Namibia is an English-speaking Southern sub-Saharan African country classified as an upper-middle-income country [31], with a Medium Human Development Index rank of 139 [29]. In 2019, the country’s age-standardised, all ages suicide rate was 13.5 per 100,000 people, higher than both the Africa rate (11.2 per 100,00 people) and global average (9.0 per 100, 000 people) [4].

Thus, the present study seeks to analyse the 2013 Namibian WHO-GSHS data on non-fatal suicidal behaviours to address the following research aims:

1. Estimate the 12-month prevalence of suicidal behaviour (ideation, planning, and attempt) among school-going adolescents 12–17 years in Namibia.
2. Describe some of the commonly reported factors at the personal/lifestyle-, family-, school-, and interpersonal relationship-levels associated with suicide ideation, planning, and (repeated) suicide attempt among school-going adolescents (aged 12–17 years) in Namibia. Considering the nature of the data analysed, his study considers suicidal behaviour to comprise suicidal ideation, planning, and attempt – excluding suicidal death.

## Methods

### Context and data source

This study drew on data from the 2013 Namibia WHO-GSHS. The survey was conducted by WHO and the Centers for Disease Control and Prevention (CDC) of the United States in collaboration with the Government of Namibia [32]. The data is publicly available and has been accessed freely for the current study from the WHO website [32].

### Study design and sampling

The WHO-GSHS is a national representative cross-sectional survey conducted to assess behavioural health factors amongst school-going adolescents (mainly aged 13 to 17) in participating WHO member countries [33]. Data was collected using a validated self-administered questionnaire consisting of items assessing a wide range of personal lifestyle (e.g., alcohol and drug use), family relationships (e.g., parental supervision and understanding), and school environment variables (e.g., truancy and bullying) [33]. Prior to the data collection, ethical approval was sought from relevant authorities as well as consent from parents/guardians of adolescents. The 2013 Namibia GSHS targeted students in grades 7–12 which is typically attended by students aged 13–17 years. A two-stage sampling approach was used for data collection. In the first stage, a cluster of schools were randomly selected from a list of all schools in Namibia using a probability proportionate to enrollment size method. This process resulted in a list of eligible and nationally representative schools. The second stage involved the random sampling of classrooms from these eligible schools, making all students in the selected classes eligible to participate. Students who volunteered to participate were then handed an anonymised computer scannable questionnaire to complete. A total of 4531 students aged 13-18years participated in the Namibia GSHS. The school response rate was 100%; that of students was 89%, while the overall response rate was 89%. The reporting of the current study was guided by the recommendations of Strengthening the Reporting of Observational Studies in Epidemiology (STROBE) [34].

### Measures

#### Outcome variables

Three domains of suicidal behaviour namely suicide ideation, planning and attempt were considered as the outcome variables for this study. The three domains were each assessed using a single-item question. Suicide ideation was measured with, “during the past 12 months, did you ever seriously consider attempting suicide?”, suicide planning with, “during the past 12 months, did you make a plan about how you would attempt suicide?” and attempt with “during the past 12 months, how many times did you actually attempt suicide?”. The responses for suicide ideation and plan were, “Yes = 1” and “No = 0” while that of attempt required students to indicate the number of times with “0”, “1”, “2 or 3”, “4 or 5”, and “6 or more times”. Guided by the GSHS recoding procedures [33], the suicide attempt variable was treated as a binary variable assigning “0” to no attempt and “1” to one or more attempts. For the purposes of examining factors responsible for repeated suicide attempts, the attempt variable was subsequently reclassified into three categories namely, no attempt, one-time attempt, and repeated attempts. Therefore, responses of adolescents who chose “1” for suicide attempt was recoded into the one-time attempt category and that of adolescents who chose “2 and above” into the repeated attempts category. Coding of variables included in this study (i.e., demographic variables, exposure factors, and missing data) are presented as supplementary material (e-Table 1).

#### Exposure variables

Participants’ demographic characteristics, mental health and lifestyle factors, interpersonal factors, school-level factors as well as family-level factors were selected as exposure variables in this study. Besides performing bivariable analyses to assess the relationships between the exposure and outcomes variables, the selection of these exposure variables was based on evidence from previous studies within sub-Saharan Africa drawing data from the WHO-GSHS [14, 27, 35–37]. Examples of the specific factors include age, school grade, gender, cannabis use, loneliness, anxiety, and alcohol use. Complete details of the variables, their groupings, survey questions and coding can be found in supplementary material (e-Table 1).

#### Statistical analyses

Reporting of the statistical analyses plan in this study is informed by the Statistical Analyses and Methods in the Published Literature (SAMPL) guidelines [38]. We limited the analysis to participants aged 12 to 17 years for two reasons: first, most of the participants were within this age range, and second, data on the precise ages below and above this age range were not available. Age and gender distribution across the analytical sample is presented as supplementary material (e-Table 2). Statistical analyses involving univariate, bivariate and multivariate testing were conducted in Stata 14.0 statistical software (StataCorp LP, College Station, Texas, USA). Prior to conducting these tests, clusters, stratification, and sample weights characteristic of data collected with complex designs were adjusted to account for possible analytical errors and make appropriate inferences [39]. Following from this, univariate analysis computing frequencies, proportions and relevant 95% confidence intervals of all study variables was done. Chi-square tests (χ^2^) of independence were performed to examine the association between the exposure and outcomes variables. Multivariate analyses with logistic regression and multinomial logistic regression were then conducted in two steps. Step 1: Logistic regression was performed to assess the sociodemographic factors and exposures variables associated with suicidal ideation, planning and attempt. Step 2: We performed a multinomial logistic model assessing the factors associated with repeated suicide attempt (keeping the base at ‘no attempt’). Given the presence of sparse data only exposure variables that reached statistical significance in the binary logistic regression models were included in the multinomial logistic model. Age, gender and school grade were included as covariates in the multivariable analyses. Statistical significance was set at an alpha of 0.05. Adjusted odds ratios (AOR) are reported for each logistic model and adjusted relative risk ratio (ARRR) for the multinomial logistic model. Given the arbitrary nature of the alpha (p < 0.05), the significance of each analysis was also determined based on CIs and their clinical importance [40, 41]. Missing responses or incomplete data on key variables were excluded from the final analysis (e-Table 1).

## Results

### Participant characteristics

A total of 3152 in-school adolescents comprising 1380 (44.3%) male and 1772 (55.7%) females were included as analytical sample in the secondary analysis for this study. The average age of these adolescents was *M* = 15.1 (*SD* = 1.4) and most of them were in grade 10–12 (60.5%). Regarding mental health and lifestyle factors, approximately 14 and 13% of adolescents had experienced loneliness and anxiety respectively and about 36% had spent 3 or more hours a day engaged in leisure time or sedentary behaviour. Additionally, about 29% and 5% had use alcohol and cannabis in the past month, respectively.

### Prevalence estimates

Table 1 presents comparable 12-month prevalence estimates of suicidal ideation (20.2% [95% CI = 18.3–22.2%]), plan (25.2% [95% CI = 22.3–28.4%]), one-time attempt (14.6% (95% CI = 12.5–16.9%]) and repeated attempt (9.9% (95% CI = 8.1–12.1%]) across the total analytic sample. Overall, 24.5% (95% CI = 20.9–28.6%) of the analytic sample reported attempted suicide during the previous 12 months. Similarly, the 12-month prevalence estimates of suicidal ideation, plan, one-time and repeated attempt were comparable between boys and girls, as the CIs of these estimates overlapped.

**Table 1:** 12-month prevalence estimates of suicidal behaviour

Suicidal behaviour	Total sample % (95%CI)	Male % (95% CI)	Female % (95%CI)
Suicide ideation	20.2 (18.3–22.2)	20.0 (17.0-23.4)	20.3 (18.4–22.4)
Suicide plan	25.2 (22.3–28.4)	25.7 (21.8–30.1)	24.8 (22.0-27.9)
One-time suicide attempt	14.6 (12.5–16.9)	15.2 (12.6–18.4)	14.0 (11.9–16.5)
Repeated suicide attempts	9.9 (8.1–12.1)	10.9 (8.8–13.6)	9.1 (7.2–11.4)
Suicide attempt (overall)	24.5 (20.9–28.6)	26.2 (21.6–31.4)	23.1 (19.7–26.9)

### Bivariate associations

As shown in Table 2, most of the exposure variables were significantly related to suicidal behaviour. Of the two demographic factors (gender, and school grade) included in the study, only, school grade was significantly related to suicidal behaviour. A high proportion of students in grade 6–9 (compared to students in grades 10–12) reported suicide ideation, plan and attempt during the previous 12 months. Among the mental health and lifestyle factors, loneliness, anxiety, alcohol use and cannabis use were all significantly related to suicidal behaviour. Leisure-time sedentary behaviour was significantly related to only suicidal ideation (χ^2^ = 4.6, df = 1, *p* = 0.01). Under the interpersonal factors, being sexually active and physical fight were related to suicide behaviour. Having a close friend was significantly related to suicide plan. Concerning school-level factors, a significant proportion of students who were physically attacked, truant and were victims of bullying reported experiences of suicidal behaviour. Some significant associations were also found among the family-related factors (e.g., intrusion of privacy by parents, food insecurity, and parental supervision) and suicidal behaviour. Notably, food insecurity and parental intrusion of privacy were both related to all the domains of suicidal behaviour examined.

**Table 2:** Bivariate analysis of the factors associated with suicidal behaviour

Study Variable	Total sample	Suicidal ideation			Suicidal plan			Suicidal attempt		
	*N* = 3152	Yes	No	χ^2^ (*p*)	Yes	No	χ^2^ (*p*)	Yes	No	χ^2^ (*p*)
	n (%)	n (%)	n (%)		n (%)	n (%)		n (%)	n (%)	
**Demographics**										
Gender				0.04 (0.87)			0.33 (0.61)			4.0 (0.08)
Male	1380 (44.3)	278 (20.0)	1102 (80.0)		362 (25.7)	1018 (74.3)		365 (26.2)	1015 (73.8)	
Female	1772 (55.7)	365 (20.3)	1407 (79.7)		457 (24.8)	1315 (75.2)		429 (23.2)	1343 (76.8)	
School grade				**11.1 (0.001)**			**47.2 (< 0.001)**			**68.8 (< 0.001)**
Grade 6–9	1918 (60.5)	423 (22.1)	1495 (77.9)		576 (29.3)	1342 (70.7)		576 (29.6)	1342 (70.4)	
Grade 10–12	1183 (39.5)	209 (17.2)	974 (82.8)		224 (18.4)	959 (81.6)		202 (16.5)	981 (83.5)	
**Mental health and lifestyle factors**										
Loneliness				**48.3 (< 0.001)**			**42.7 (< 0.001)**			**44.7 (< 0.001)**
Yes	449 (14.3)	146 (32.3)	303 (67.7)		171 (37.7)	278 (62.3)		170 (37.0)	279 (63.0)	
No	2673 (85.8)	488 (18.1)	2185 (81.9)		643 (23.2)	2030 (76.8)		614 (22.3)	2059 (77.7)	
Worry/anxiety				**38.7 (< 0.001)**			**50.0 (< 0.001)**			**51.4 (< 0.001)**
Yes	435 (13.8)	138 (31.3)	297 (68.8)		169 (39.0)	266 (61.0)		167 (38.2)	268 (61.8)	
No	2699 (86.2)	499 (18.3)	2200 (81.7)		647 (23.1)	2052 (76.9)		620 (22.2)	2079 (77.8)	
Alcohol use				**8.6 (0.01)**			**4.9 (0.02)**			**3.6 (0.04)**
Yes	860 (29.3)	204 (23.4)	656 (76.6)		247 (28.0)	613 (72.0)		234 (26.6)	626 (73.4)	
No	2120 (70.7)	400 (18.7)	1720 (81.3)		531 (24.2)	1589 (75.8)		511 (23.3)	1609 (76.7)	
Leisure-time sedentary behaviour				**4.6 (0.01)**			5.2 (0.06)			1.5 (0.29)
Yes	1138 (36.4)	248 (21.9)	890 (78.1)		320 (27.4)	818 (72.6)		301 (25.5)	837 (74.5)	
No	1966 (63.6)	377 (18.7)	1589 (81.3)		483 (23.8)	1483 (76.2)		475 (23.6)	1491 (76.4)	
Cannabis use				**47.4 (< 0.001)**			**25.9 (0.001)**			**60.4 (< 0.001)**
Yes	134 (4.6)	58 (42.4)	76 (57.6)		59 (42.8)	75 (57.2)		72 (50.8)	62 (49.2)	
No	2909 (95.4)	552 (18.7)	2357 (81.3)		715 (23.8)	2194 (76.2)		668 (22.3)	2241 (77.7)	
**Interpersonal factors**										
Being sexually active				**17.9 (0.001)**			**16 (0.004)**			**10.4 (0.01)**
Yes	958 (33.5)	228 (24.0)	730 (76.0)		456 (22.2)	1495 (77.8)		260 (25.6)	698 (73.4)	
No	1951 (66.5)	349 (17.4)	1602 (82.6)		279 (29.1)	679 (70.9)		431 (21.2)	1520 (78.8)	
Close friends				2.1 (0.20)			**7.4 (0.03)**			0.2 (0.71)
None	403 (13.0)	93 (22.7)	310 (77.3)		125 (30.6)	278 (69.5)		105 (25.1)	298 (74.9)	
One or more	2701 (87.0)	536 (19.7)	2165 (80.3)		677 (24.2)	2024 (75.8)		670 (24.2)	2031 (75.8)	
Physical fight				28.3 (< 0.001)			**67.7 (< 0.001)**			**127.7 (< 0.001)**
Yes	1074 (34.4)	274 (25.5)	800 (74.5)		378 (34.1)	696 (65.9)		402 (36.4)	672 (63.6)	
No	2065 (65.6)	366 (17.4)	1699 (82.6)		438 (20.6)	1627 (79.4)		358 (18.1)	1680 (81.9)	
**School-level factors**										
Physical attack				**51.3 (< 0.001)**			**81.2 (< 0.001)**			**184.6 (< 0.001)**
Yes	1253 (39.8)	327 (26.5)	926 (73.5)		429 (33.8)	824 (66.2)		472 (37.4)	781 (62.6)	
No	1859 (60.2)	308 (16.0)	1551 (84.0)		379 (19.5)	1480 (80.5)		313 (16.0)	1546 (84.0)	
Truancy				**23.0 (0.001)**			**65.5 (< 0.001)**			**84.7 (< 0.001)**
Yes	747 (24.0)	193 (26.2)	554 (73.8)		273 (36.5)	474 (63.5)		279 (36.9)	468 (36.1)	
No	2355 (76.0)	436 (18.1)	1919 (81.9)		535 (21.7)	1820 (78.3)		497 (20.3)	1858 (79.7)	
Peer support				0.1 (0.78)			2.1 (0.27)			0.5 (0.52)
Yes	853 (27.1)	179 (20.6)	674 (79.4)		239 (26.9)	614 (73.1)		223 (25.2)	630 (74.8)	
No	2270 (72.9)	459 (20.0)	1811 (80.0)		570 (24.4)	1700 (75.6)		558 (24.0)	1712 (76.0)	
Bullying victimisation				**36.5 (< 0.001)**			**60.5 (< 0.001)**			**144.0 (< 0.001)**
Yes	1311 (44.7)	326 (25.3)	985 (74.7)		415 (31.8)	896 (68.2)		448 (34.1)	863 (65.9)	
No	1577 (55.4)	264 (16.3)	1313 (83.7)		319 (19.2)	1258 (80.8)		247 (15.0)	1330 (85.0)	
**Family-level factors**										
Parental supervision				1.3 (0.40)			**23.1 (0.001)**			**17.5 (0.01)**
Yes	1369 (42.9)	290 (21.0)	1079 (79.0)		412 (29.3)	957 (70.7)		389 (28.1)	980 (72.0)	
No	1743 (57.1)	342 (19.3)	1401 (80.7)		392 (21.8)	1351 (78.2)		389 (21.5)	1354 (78.5)	
Parental understanding				4.9 (0.07)			2.3 (0.26)			0.1 (0.71)
Yes	1288 (40.9)	238 (18.2)	1050 (81.8)		314 (23.6)	974 (76.4)		315 (23.8)	973 (76.2)	
No	1794 (59.1)	389 (21.4)	1405 (78.6)		482 (26.0)	1312 (74.0)		450 (24.4)	1344 (75.6)	
Parental monitoring				1.0 (0.33)			0.1 (0.86)			**6.3 (0.03)**
Yes	1104 (35.1)	214 (19.1)	890 (80.9)		290 (24.8)	814 (75.2)		253 (21.7)	851 (78.3)	
No	2018 (64.9)	420 (20.6)	1598 (79.4)		516 (25.2)	1502 (74.8)		529 (25.8)	1489 (74.3)	
Parental intrusion of privacy				**16.0 (0.002)**			**19 (0.002)**			**41.1 (< 0.001)**
Yes	1764 (56.7)	313 (17.5)	1451 (82.5)		401 (22.1)	1363 (77.9)		363 (20.0)	1401 (80.0)	
No	1356 (43.3)	319 (23.3)	1037 (76.7)		405 (29.0)	951 (71.0)		416 (30.0)	940 (70.0)	
Food insecurity				**13.9 (0.001)**			**22.3 (0.001)**			**38.3 (< 0.001)**
Yes	310 (9.9)	87 (28.3)	223 (71.7)		113 (36.3)	197 (63.7)		119 (38.8)	191 (61.2)	
No	2796 (90.1)	546 (19.3)	2250 (80.7)		692 (24.0)	2104 (76.0)		659 (22.8)	2137 (77.2)	

*Note*: Statistically significant results are in bold face

### Multivariate associations

Tables 3 and 4 show the findings of the adjusted logistic regression models and multinomial models respectively.

**Table 3:** Multivariate logistic regression predicting factors on suicidal behaviour

Variable	Suicide Ideation (N = 2199)			Suicide Plan (N = 2199)			Suicide Attempt (N = 2199)		
	β	AOR [95% CI]	*p*-value	β	AOR [95% CI]	*p*-value	β	AOR [95 CI]	*p*-value
**Demographics**									
Gender:									
Female		1			1			1	
Male	-0.15	0.86 [0.64, 1.15]	0.309	-0.19	0.82 [0.66, 1.03]	0.088	-0.09	0.91 [0.77, 1.08]	0.269
Age	**0.12**	**1.13 [1.04, 1.22]**	**0.004**	0.10	1.11 [0.99, 1.23]	0.061	**0.10**	**1.10 [1.00, 1.21]**	**0.047**
School grade:									
Grade 6–9		1			1			1	
Grade 10–12	**-0.41**	**0.66 [0.52, 0.85]**	**0.002**	**-0.64**	**0.52 [0.40, 0.70]**	**< 0.001**	**-0.72**	**0.49 [0.35, 0.69]**	**< 0.001**
**Mental health and lifestyle factors**									
Loneliness	**0.54**	**1.71 [1.22, 2.39]**	**0.003**	**0.54**	**1.71 [1.27, 2.29]**	**0.001**	**0.47**	**1.60** **[1.17, 2.18]**	**0.005**
Worry/anxiety	**0.40**	**1.49** **[1.13, 1.96]**	**0.007**	**0.44**	**1.56 [1.11, 2.18]**	**0.013**	0.31	1.36 [0.94, 1.97]	0.104
Alcohol use	0.15	1.16 [0.81, 1.66]	0.389	0.17	1.19 [0.91, 1.54]	0.189	0.05	1.05 [0.76, 1.46]	0.769
Leisure-time sedentary behaviour	**0.32**	**1.36 [1.08, 1.72]**	**0.009**	0.22	1.25 [0.96, 1.63]	0.090	0.10	1.11 [0.83, 1.49]	0.479
Cannabis use	0.35	1.42 [0.84, 2.42]	0.182	0.48	1.61 [0.99, 2.64]	0.057	**0.78**	**2.18** **[1.29, 3.67]**	**0.005**
**Interpersonal factors**									
Being sexually active	0.20	1.22 [0.93, 1.60]	0.147	0.30	1.34 [0.99, 1.81]	0.054	0.18	1.20 [0.95, 1.53]	0.125
Close friends	0.08	1.08 [0.72, 1.63]	0.694	-0.14	0.87 [0.58, 1.31]	0.480	0.06	1.06 [0.73, 1.54]	0.745
Physical fight	0.18	1.19 [0.84, 1.70]	0.309	**0.30**	**1.35 [1.00, 1.83]**	**0.048**	0.21	1.24 [0.93, 1.65]	0.141
**School-level factors**									
Physical attack	**0.42**	**1.53 [1.15, 2.03]**	**0.005**	**0.41**	**1.51 [1.15, 1.99]**	**0.005**	**0.69**	**1.99 [1.56, 2.56]**	**< 0.001**
Truancy	-0.05	0.96 [0.68, 1.35]	0.788	0.19	1.21 [0.88, 1.66]	0.236	0.17	1.19 [0.83, 1.70]	0.338
Peer support	0.07	1.08 [0.81, 1.44]	0.609	0.25	1.28 [0.96, 1.72]	0.091	0.20	1.22 [0.96, 1.54]	0.098
Bullying victimisation	0.18	1.20 [0.93, 1.54]	0.157	**0.35**	**1.42 [1.12, 1.79]**	**0.005**	**0.61**	**1.83 [1.39, 2.42]**	**< 0.001**
**Family-level factors**									
Parental supervision	0.07	1.06 [0.82, 1.40]	0.619	**0.38**	**1.46 [1.12, 1.90]**	**0.006**	0.26	1.29 [0.98, 1.70]	0.069
Parental understanding	-0.26	0.77 [0.56, 1.06]	0.106	-0.21	0.81 [0.59, 1.12]	0.202	0.16	1.17 [0.92, 1.48]	0.187
Parental monitoring	-0.07	0.93 [0.76, 1.14]	0.478	-0.04	0.96 [0.72, 1.27]	0.751	**-0.32**	**0.73 [0.57, 0.94]**	**0.016**
Parental intrusion of privacy	**0.29**	**1.34 [1.04, 1.73]**	**0.024**	**0.25**	**1.29 [1.00, 1.65]**	**0.047**	**0.44**	**1.55 [1.21, 1.99]**	**0.001**
Food insecurity	0.23	1.26 [0.89, 1.78]	0.181	-0.07	0.93 [0.71, 1.23]	0.602	0.15	1.16 [0.82, 1.65]	0.392
McFadden’s *R*^2^		0.054			0.08			0.110	
McKelvey and Zavoina’s *R*^2^		0.090			0.14			0.182	
Hosmer–Lemeshow GOF test (sig.)		2072 (0.30)			2064 (0.35)			2011 (0.67)	
Overall percentage correctly classified		81.40%			76.40%			79.45%	

*Note*. AOR = adjusted odds ratio; CI = Confidence Interval; statistically significant results are in bold face

**Table 4:** Multinomial logistic regression predicting factors on repeated suicide attempt (N = 2660)

Variable	One-time attempt			Repeated attempts		
	β	ARRR [95% CI]	*p*-value	β	ARRR [95 CI]	*p*-value
**Demographics**						
Gender:						
Female		1			1	
Male	-0.02	0.98 [0.80, 1.20]	0.849	0.11	1.12 [0.86, 1.45]	0.395
Age	**0.12**	**1.13 [1.03, 1.24]**	**0.013**	0.10	1.10 [0.96, 1.27]	0.164
School grade:						
Grade 6–9		1			1	
Grade 10–12	**-0.69**	**0.50 [0.36, 0.70]**	**< 0.001**	**-0.67**	**0.51 [0.35, 0.75]**	**0.001**
**Mental health and lifestyle factors**						
Loneliness	**0.43**	**1.54 [1.05, 2.25]**	**0.028**	**0.72**	**2.06 [1.27, 3.35]**	**0.005**
Cannabis use	0.26	1.29 [0.65, 2.58]	0.455	**1.31**	**3.72 [2.08, 6.65]**	**< 0.001**
**School-level factors**						
Physical attack	**0.65**	**1.92 [1.47, 2.50]**	**< 0.001**	**1.07**	**2.92 [2.28, 3.74]**	**< 0.001**
Bullying victimisation	**0.52**	**1.68 [1.27, 2.22]**	**0.001**	**0.96**	**2.60 [1.83, 3.70]**	**< 0.001**
**Family-level factors**						
Parental monitoring	-0.22	0.80 [0.61, 1.05]	0.108	-0.20	0.82 [0.57, 1.18]	0.269
Parental intrusion of privacy	**0.35**	**1.41 [1.13, 1.76]**	**0.003**	**0.58**	**1.78 [1.30, 2.44]**	**0.001**

*Note*. ARRR = adjusted relative risk ratios; CI = Confidence Interval; statistically significant results are in bold face.

### Logistic regression

As presented in Table 3, the most important exposure factors contributing to the increased odds of all the domains of suicide included grade in school, loneliness, physical attack and parental intrusion of privacy. Other variables including age, anxiety, leisure-time sedentary behaviour, cannabis use, physical fight, bullying victimisation, and parental monitoring were related to at least one form of suicide behaviour. For instance, sedentary behaviour was associated with increased the odds of only suicidal ideation (AOR = 1.36, 95% CI:1.08, 1.72, *p* = 0.009), while parental monitoring was related to reduced odds of attempted suicide (AOR = 0.73, 95% CI: 0.57, 0.94, *p* = 0.001). Interestingly, although gender, alcohol use, being sexually active, number of close friends, truancy, peer support, parental understanding, and food insecurity were associated with each of the suicidal behaviour outcomes, none of these associations reached the desired threshold of statistical significance (Table 3).

### Multinomial logistic regression

As shown in Table 4, five factors (physical attack victimisation, bullying victimisation, loneliness, parental intrusion of privacy, and grade in school) were associated with the likelihood of both one-time attempt and repeated attempted suicide during the previous 12 months. Specifically, physical attack victimisation, bullying victimisation, loneliness, and parental intrusion of privacy were associated with increased likelihood of both one-time attempt and repeated attempted suicide. However, adolescents in grades 10–12 (compared to those in grades 6–9) had reduced relative risk of reporting one-time attempt (ARRR = 0.50, 95% CI: 0.36, 0.70, *p* < 0.001) or repeated attempt (ARRR = 0.51, 95% CI:0.35, 0.75, *p* = 0.001). It is also worthy of mention that while the likelihood association between cannabis use and one-time suicide attempt did not reach the desired threshold of statistical significance, comparatively, the likelihood association between cannabis use and repeated suicide attempt showed the strongest statistical and clinical importance (ARRR = 3.72, 95% CI: 2.08, 6.65, *p* < 0.001) between the two statistical models. Put differently, students who used cannabis were at about three times increased relative risk of repeated attempted suicide during the previous 12 months, compared to students who did not use cannabis.

## Discussion

This cross-sectional study sought to describe the 12-month prevalence estimates of suicidal behaviour (ideation, planning, and [one-time and repeated] attempts) and associated factors among school-going adolescents aged 12–17 years in Namibia, drawing on the 2013 Namibian WHO-GSHS. In summary, the current study has two key findings. First, comparable estimates of suicidal behaviour were found between boys and girls, with about 2 in 10 adolescents reporting suicidal ideation, planning, or attempt in the previous 12 months. Approximately, 1 in 20 students reported repeated attempted suicide during the previous 12 months. Secondly, physical attack victimisation, bullying victimisation, loneliness, and parental intrusion of privacy were associated with increased likelihood of suicidal ideation, planning, one-time suicide attempt and repeated attempted suicide. Adolescents in higher grades (grades 10–12), compared to those in lower grades (grades 6–9), had reduced relative risk of reporting one-time attempt or repeated attempts at suicide. Cannabis use was associated with increased relative risk of repeated attempted suicide.

The 12-month prevalence estimates of suicidal behaviour found in the current study are comparable to those found generally among school-going adolescents within the (sub-Saharan) African region, where estimates of suicidal ideations, planning and attempt range from 20.1 to 29% [7–9]. Beyond the similarity of the estimates of this study to those reported earlier from other Southern African countries – e.g., Eswatini, Malawi, Mozambique, and South Africa – [14, 19, 21, 23, 24], the evidence regarding comparable estimates between boys and girls have also been reported from other Southern African countries and sub-Saharan Africa in general [10, 21]. The cross-national similarity of the estimates is to be expected, considering that the data are drawn from the WHO-GSHS conducted in the respective countries using the same measures and definitions of included variables. The lack of significant gender differences in the estimates of suicidal behaviour could be pointing to the possibility that, perhaps, the factors presenting as risks are comparably difficult for both school-going boys and girls in Namibia. This finding could also be supporting the emerging evidence that self-harm and suicidal behaviour are not neatly differentiated between boys and girls within sub-Saharan Africa [10]. Taken together, this evidence could be underscoring the importance of universal intervention and prevention efforts focused on suicidal behaviour in both school-going adolescent boys and girls in Namibia.

We found physical attack victimisation, bullying victimisation, loneliness, and parental intrusion of privacy to be significantly associated with increased likelihood of suicidal ideation, planning, one-time suicide attempt and repeated attempted suicide. Cannabis use was associated with increased relative risk of (repeated) attempted suicide. Global and regional systematic reviews and meta-analyses [7, 8, 10, 12, 42] and recent primary studies drawing data mainly from the WHO-GSHS [14, 21, 23, 36, 37, 43, 44] have also identified these factors to be critical in school-going adolescent suicidal tendencies and behaviours.

Further, within the lens of the socio-ecological model, our finding supports the multi-factorial, multi-layered and multi-contextual nature of the factors associated with suicidal behaviour among adolescents [15, 16]. In broader terms, the identified key associated factors of suicidal behaviour among adolescents in the current study (i.e., physical attack victimisation, bullying victimisation, and parental intrusion of privacy) support evidence in the literature that exposure to (longstanding) interpersonal social adversities and relational difficulties occurring in the family, school or within peer relationships contribute to suicidal behaviour among young people [10, 45, 46]. Besides being a common phenomenon, bullying victimisation has a strong positive association with involvement physical fighting and other interpersonal adversities among school-going adolescents in Namibia [47, 48]. Considered as an antecedent of suicidal thoughts and behaviour, social adversities (e.g., physical attack victimisation, and bullying victimisation) result in internalising problems, often leading to lowered self-esteem, self-blame, and self-dislike, which in turn heighten the vulnerability to self-harming thoughts and behaviours in adolescents [49].

Recent literature is replete with evidence that in both high-income countries and LMICs, lifestyle factors, particularly health risk behaviours (e.g., cannabis smoking, alcohol use) and – untreated – mental health problems (e.g., loneliness, anxiety, depression) have strong association with suicidal behaviour among adolescents[11, 42, 44, 50–52]. Although cannabis possession and use are illegal in Namibia, national-level estimates suggest that among school-going adolescents 6.6% boys and 4% girls report having used cannabis during the previous month [53] – a clear indication that there are lapses and problems in the implementation of the law [54]. What is not readily clear from the current study is whether the participants’ cannabis use was a coping strategy for their adverse mental health experiences (e.g., loneliness or anxiety) or for another purpose. Nonetheless, there is evidence to suggest that substance (cannabis or alcohol) use has negative – mental – health outcomes among adolescents. Besides underlying increased susceptibility to brain damage in the long-term, cannabis use impairs judgement and memory, increases impulsivity and negative mood, and potentially complicates the natural course of loneliness, anxiety, and depression – which in turn elevate the risk for suicidal thoughts and behaviour among adolescents [51, 55–58].

Our finding that school-going adolescents in higher grades, compared to those in lower grades, have reduced relative risk of reporting one-time or repeated attempts at suicide supports earlier evidence from South Africa [59], but it is inconsistent with a recent finding from Ghana, where no significant association was observed between school grade and suicidal behaviour [60]. Whereas explanations for this school grade difference are not readily clear from the Southern African context, perhaps, in Namibia, curriculum-based functional mental health literacy could be suggested. Among other aims, the Namibian school curriculum seeks “to foster the highest moral, ethical and spiritual values such as integrity, responsibility, equality, and reverence for life” [30]. Maybe, the value of ‘reverence for life’ – which essentially proscribes and eschews suicidal thoughts and actions [61] – might have been more actionably consolidated in students in upper school grades than those in lower grades. Beyond this speculation, further studies are needed to understand this differentiation of suicidal thoughts and behaviours in terms of school grade in Namibia but also within the general (Southern) sub-Saharan African context.

While the factors showing significant associations with suicidal behaviour (including repeated attempted suicide) are identified, it is also imperative to comment on the (exposure) variables reported to be important in the literature but showed no significant associations with the outcomes in the current study. Interestingly, in the current study, although alcohol use, being sexually active, number of close friends, truancy, peer support, parental understanding, and food insecurity were associated with each of the suicidal behaviour outcomes, none of these associations reached the desired threshold of statistical significance. While we suspect sparse data to account for the lack of statistical significance, we believe future studies involving relatively large responses to each of these data items will contribute to clarifying the statistical and clinical significance of their associations with school-going adolescent suicidal behaviour in Namibia.

This study has identified the key factors associated with adolescent suicidal behaviours to exist mainly at the individual level/ (e.g., loneliness, anxiety, cannabis use), within school (e.g., bullying victimisation, school grade), family (e.g., intrusion of privacy by parents, parental monitoring), and community context (e.g., physical attack victimisation). The implication of the multi-ecological nature of the key evidence for practice is that intervention and prevention efforts must be designed with a multi-sectoral and multi-layered orientation. For example, while the initiation (or improvement of existing) anti-alcohol and substance use and anti-bullying polices are needed to enhance school social climate, community-level training for supportive parenting can be designed for families with adolescents. Similarly, while parents and significant others living with adolescents need to be observant regarding the identification of warning signs of potential adolescent suicidal behaviours, the Namibian Ministry of Education could consider including mental health literacy and help-seeking lessons in the curriculum for high schools – this would contribute to improving help-seeking and self-care behaviours among school-going adolescents at risk of self-harm and suicidal tendencies and other emotional crises, including loneliness, anxiety and depression.

### Strengths and Limitations

A critical significance of this study is that it contributes to addressing the problem of dearth research on suicidal behaviour among adolescents in Namibia. Data on adolescent mental health (outcomes) are still insufficient to inform policy, intervention efforts, and prevention programmes in Namibia [62]. In particular, data on self-harm and suicidal behaviours among (both school-going and out-of-school) adolescents in Namibia are less than enough [10, 63]. Additionally, this study contributes broadly to advance our knowledge and understanding of the prevalence and associated factors of suicidal behaviour among in-school adolescents in Namibia in that the study draws on a relatively large data accessed from a nationally representative sample.

Beyond these strengths, there are noteworthy limitations of this study. The findings of the study should be generalised with caution, as the key evidence may not necessarily apply to out-of-school adolescents. While the study sample excludes students who were absent on the day of the survey, evidence suggests that the average annual dropout rate in Namibia ranges between 3 and 10.4% [64]. The one-time cross-sectional survey design used for the WHO-GSHS implies that the outcome and exposure variables were measured at the same time point, making it impossible to identify sequence, temporal link, and causal relationship between the exposure and outcome variables. Thus, consistent with recommendations by recent studies from of other countries within the continent [10, 13, 65], future studies using more robust approaches, including longitudinal designs and carefully designed qualitative studies are needed for contextually nuanced understanding of self-harm and suicidal behaviours among adolescents in Namibia and (sub-Saharan) Africa generally.

Notably, in the current study, it is not clear why the estimates of suicidal planning and attempt are higher than suicidal ideation. Similar evidence has been reported from other sub-Saharan African countries drawing on the WHO-GSHS data – e.g., Benin, Ghana, Liberia, Malawi, and Sierra Leone [21, 37, 66–68]. However, this evidence is unconventional, counterintuitive, and not in keeping with the suicide process or pathway model [69, 70], which superlatively suggests that, typically, estimates of ideation are expected to be highest, followed by estimates of planning, then estimates of attempt. Perhaps, the use of a single-item measures in the WHO-GSHS to assess these outcomes could account for this unconventional finding. As cautioned elsewhere, the use of single-item measure to assess suicidal ideation, planning, and attempt must be interpreted cautiously, as single-item measures typically result in misclassification of suicidal behaviours and higher estimates [71]. Specially, we also suspect that the lower estimate of suicidal ideation – relative to suicidal attempt – in the current study may be due to impulsivity. There is evidence from high-income countries to suggests that impulsivity may result in the onset of suicidal attempt among adolescents, even in the absence of prior suicidal ideation [72, 73].

## Conclusion

The evidence of this study adds to the literature on non-fatal suicidal behaviour among school-going adolescents in (sub-Saharan) Africa, but also underscores the marked estimates of suicide ideation, planning, and (repeated) suicide attempt among school-going adolescents (aged 12–17 years) in Namibia. The relatively high prevalence estimates and multi-layered correlates (at the individual-level, family-level, interpersonal-level, school context and the broader community context) contribute to our understanding of adolescent suicide in Namibia. The evidence highlights the importance of paying more attention to addressing the mental health needs (including those related to psychological and social wellness) of school-going adolescents in Namibia. While the current study suggests that further research is warranted to explicate the pathways to adolescent suicide in Namibia, identifying and understanding the correlates of adolescent suicidal ideations and non-fatal suicidal behaviours are useful for intervention and prevention programmes.

## Electronic supplementary material

Below is the link to the electronic supplementary material.

Supplementary Material 1

## Data Availability

The datasets used and/or analysed during the current study are freely available from the WHO website: https://extranet.who.int/ncdsmicrodata/index.php/catalog/478. The 2013 Namibia GSHS questionnaire is also available freely on the WHO’s website: https://extranet.who.int/ncdsmicrodata/index.php/catalog/478#metadata-questionnaires.

## Abbreviations

CDC: Centers for Disease Control and Prevention
GSHS: Global School-based Student Health Survey
LMICs: Low- and middle-income countries
WHO: World Health Organization
