# Supplementary material for: Adolescent suicidal behaviour in Namibia: a cross-sectional study of prevalence and correlates among 3,152 school learners aged 12–17 years
Source: BMC Psychiatry. 2023 Mar 15;23:169. doi: 10.1186/s12888-023-04646-7 (PMC10018865; doi:10.1186/s12888-023-04646-7)
Supplement: Supplementary file 1 — Supplementary Material 1 [file 12888_2023_4646_MOESM1_ESM.docx]

**Supplementary Material**

e-Table 1: Coding of demographic variables and exposure factors included study, and missing data

| **Study Variables** | Survey questions and coding | N = 4531; missing = freq. (%) |
| --- | --- | --- |
| **Outcome variables** |  |  |
| **Suicide Ideation** | During the past 12 months, did you ever seriously consider attempting suicide? | N = 3152, Missing = 0 |
| Yes | Yes = 1 |  |
| No | No = 0 |  |
| **Suicide Plan** | During the past 12 months, did you make a plan about how you would attempt suicide? | N = 3152, Missing = 0 |
| Yes | Yes = 1 |  |
| No | No = 0 |  |
| **Suicide Attempt** | During the past 12 months, how many times did you actually attempt suicide? | N = 3152, Missing = 0 |
| Yes | 1 time to 6 or more times=1 |  |
| No | 0 times = 0 |  |
| **Demographics** |  |  |
| **Gender** | What is your sex? | N = 3152, Missing = 0 |
| Male | Male=1 |  |
| Female | Female=0 |  |
| **Age** | How old are you? | N = 3152, Missing = 0 |
| **School Grade** | In what grade are you? | N = 3101, Missing = 51 (1.62) |
| Grade 6-9 | Grade 6 to 9=0 |  |
| Grade 10-12 | Grade 10 to 12=1 |  |
| **Mental health and lifestyle factors** |  |  |
| **Loneliness** | During the past 12 months, how often have you felt lonely? | N = 3122, Missing = 30 (0.95) |
| Yes | Most of the time/Always=1 |  |
| No | Never/Rarely/Sometimes=0 |  |
| **Worry/Anxiety** | During the past 12 months, how often have you been so worried about something that you could not sleep at night? | N = 3134, Missing = 18 (0.57) |
| Yes | Most of the time/Always=1 |  |
| No | Never/Rarely/Sometimes=0 |  |
| **Alcohol use** | During the past 30 days, on how many days did you have at least one drink containing alcohol? | N = 2980, Missing = 172 (5.46) |
| Yes | 1or 2 times to All 30 days=1 |  |
| No | 0 times=0 |  |
| **Leisure-time Sedentary behaviour** | How much time do you spend during a typical or usual day sitting and watching television, playing computer games, talking with friends, or doing other sitting activities such as playing cards, dominos, chess, and scrabble. | N = 3104, Missing = 48 (1.52) |
| Yes | 3-4 hours to more than 8 hours per day=1 |  |
| No | Less than 1 hour per day/1 to 2 hours per day=0 |  |
| **Cannabis use** | During the past 30 days, how many times have you used marijuana (also called dagga, weed, boom, cannibus, stop, grass, pipt, stop, and joint)? | N = 3043, Missing = 109 (3.46) |
| Yes | 1-2 times to 20 or more times=1 |  |
| No | 0 times=0 |  |
| **Interpersonal factors** |  |  |
| **Sexual experience** | During your life, with how many people have you had sexual intercourse? | N = 2909, Missing = 243 (7.71) |
| Yes | 1 person to 6 or more people=1 |  |
| No | I have never had sexual intercourse=0 |  |
| **Close Friends** | How many close friends do you have? | N = 3104, Missing = 48 (1.52) |
| One or more | 1 friend to 3 or more friends=1 |  |
| None | 0 friends=0 |  |
| **Physical fight** | During the past 12 months, how many times were you in a physical fight? | N = 3139, Missing = 13 (0.41) |
| Yes | 1 time to 12 or more times=1 |  |
| No | 0 times=0 |  |
| **School-level factors** |  |  |
| **Physical Attack** | During the past 12 months, how many times were you physically attacked? | N = 3112, Missing = 40 (1.27) |
| Yes | 1 time to 12 or more times=1 |  |
| No | 0 times=0 |  |
| **Truancy** | During the past 30 days, on how many days did you miss classes or school without permission? | N = 3102, Missing = 50 (1.59) |
| Yes | 1 day to 10 or more days=1 |  |
| No | 0 days=0 |  |
| **Peer Support** | During the past 30 days, how often were most of the students in your school kind and helpful? | N = 3123, Missing = 29 (0.92) |
| Yes | Most of the time/Always=1 |  |
| No | Never/Rarely/Sometimes=0 |  |
| **Bullying Victimisation** | During the past 30 days, on how many days were you bullied? | N = 2888, Missing = 264 (8.38) |
| Yes | 1 or 2 days to All 30 days=1 |  |
| No | 0 days=0 |  |
| **Family factors** |  |  |
| **Parental Supervision** | During the past 30 days, how often did your parents or guardians check to see if your homework was done? | N = 3112, Missing = 40 (1.27) |
| Yes | Most of the time/Always=1 |  |
| No | Never/Rarely/Sometimes=0 |  |
| **Parental understanding** | During the past 30 days, how often did your parents or guardians understand your problems and worries? | N = 3082, Missing = 70 (2.22) |
| Yes | Sometimes/Most of the time/Always=1 |  |
| No | Never/Rarely/Sometimes=0 |  |
| **Parental monitoring** | During the past 30 days, how often did your parents or guardians really know what you were doing with your free time? | N = 3122, Missing = 30 (0.95) |
| Yes | Sometimes/Most of the time/Always=1 |  |
| No | Never/Rarely/Sometimes=0 |  |
| **Parental intrusion of privacy** | During the past 30 days, how often did your parents or guardians go through your things without your approval? | N = 3120, Missing = 32 (1.02) |
| Yes | Sometimes/Most of the time/Always=1 |  |
| No | Never/Rarely=0 |  |
| **Food insecurity** | During the past 30 days, how often did you go hungry because there was not enough food in your home? | N = 3106Missing = 46 (1.46) |
| Yes | Most of the time/Always=1 |  |
| No | Never/Rarely/Sometimes =0 |  |

e-Table 2: Final eligible sample with complete data included in analysis (n = 3152)

|  | Total  3152 (100%) | Male  1380 (43·8%) | Female  1772 (56·2%) |
| --- | --- | --- | --- |
| Age (in years) | n (%) | n (%) | n (%) |
| 12 | 59 (1·9) | 20 (1·4) | 39 (2·2) |
| 13 | 492 (15·6) | 170 (12·3) | 322 (18·2) |
| 14 | 562 (17·8) | 231 (16·7) | 331 (18·7) |
| 15 | 712 (22·6) | 331 (24·0) | 381 (21·5) |
| 16 | 663 (21·0) | 307 (22·2) | 356 (20·1) |
| 17 | 664 (21·1) | 321 (23·3) | 343 (19·4) |
